# Endolymphatic Ethiodized Oil Intranodal Lymphangiography and Cyanoacrylate Glue Embolization for the Treatment of Postoperative Lymphatic Leak After Robot-Assisted Laparoscopic Pelvic Resection

**DOI:** 10.1089/cren.2018.0026

**Published:** 2018-05-01

**Authors:** Hannah Hill, Ravi N. Srinivasa, Joseph J. Gemmete, Anthony Hage, Jacob Bundy, Jeffrey Forris Beecham Chick

**Affiliations:** ^1^ Department of Radiology, Division of Vascular and Interventional Radiology, University of Michigan Health System, Ann Arbor, Michigan.; ^2^ Cardiovascular and Interventional Radiology, Inova Alexandria Hospital, Alexandria, Virginia.

**Keywords:** ethiodized oil intranodal lymphangiography, endolymphatics, cyanoacrylate glue, chylous ascites, lymphoceles, urologic surgery

## Abstract

***Purpose:*** To report the approach, technical success, clinical outcomes, complications, and follow-up of ethiodized oil intranodal lymphangiography with cyanoacrylate glue embolization for the treatment of lymphatic leak after robot-assisted laparoscopic pelvic resection.

***Materials and Methods:*** Four men with mean age 68.7 ± 14.3 years were treated with ethiodized oil intranodal lymphangiography with cyanoacrylate embolization for postoperative lymphatic leak. Patients underwent either (1) cystoprostatectomy with ileal conduit and bilateral extensive pelvic lymph node dissection for muscle-invasive urothelial carcinoma and presented with postoperative lymphatic ascites (*n* = 2) or (2) prostatectomy with bilateral standard pelvic lymph node dissection for prostate carcinoma and presented with postoperative pelvic lymphoceles (*n* = 2). Intranodal lymphangiography and embolization procedural details, technical success, clinical outcomes, and follow-up were recorded.

***Results:*** In four patients, a total of six ethiodized oil intranodal lymphangiograms were performed, two procedures being repeated interventions. Inguinal lymph node catheterization and ethiodized oil lymphangiography was technically effective in all procedures. A mean of 5.2 ± 2.0 mL of ethiodized oil was used for lymphatic opacification. Cyanoacrylate was diluted to 24.2% with ethiodized oil and 0.44 mL of cyanoacrylate was instilled during first time interventions. On repeat procedures, cyanoacrylate was diluted to 51.7%, and 0.52 mL was instilled. The primary clinical success rate was 50% (*n* = 2/4). Clinical success was achieved in all patients after two interventions (*n* = 4; 100%). No complications were reported at mean follow-up of 134.7 ± 79.2 days (range: 59–248 days).

***Conclusion:*** Ethiodized oil intranodal lymphangiography with direct cyanoacrylate glue embolization is a minimally invasive treatment option for lymphatic leak after pelvic resection.

## Introduction

Disruption of lymphatics during genitourinary procedures may lead to clinically significant lymphatic leaks precipitating the development of lymphoceles or accumulation of ascites. Rates of postoperative lymphatic complications requiring interventional treatment following radical prostatectomy and pelvic lymphadenectomy are reportedly as high as 15%.^1^ Persistent lymphatic leak may complicate postoperative recovery with pain from pelvic nerve compression, increased infection rates, delayed wound healing, nutritional deficiencies, and, ultimately, prolonged hospital stays.^1^

Lymphangiography with endolymphatic cyanoacrylate embolization has diagnostic and therapeutic potential by decreasing the rate of fluid accumulation in cases of persistent lymphatic leak. Lymphangiography with ethiodized oil allows for the anatomic localization of pelvic and retroperitoneal lymphatic leaks and, by virtue of an *in vivo* reactive fibrotic response, has some, therapeutic potential.^2,3^ Additional embolization with endolymphatic instillation of *n*-butyl cyanoacrylates is commonly used to therapeutically facilitate occlusion of lymphatic disruptions.^2,3^ Approaches to endolymphatic embolization via the “closest upstream lymph node”^4,5^ and “direct upstream lymphatic”^4,6^ have been described and proven therapeutically efficacious. These approaches; however, require gaining additional nodal or lymphatic vessel access following the completion of diagnostic lymphangiography. Only one report to date outlines the feasibility, demonstrated in one case, of direct cyanoacrylate instillation via the same nodal access site used for intranodal lymphangiography.^7^

The purpose of this report is to describe the approach, technical success, clinical outcomes, complications, and follow-up of ethiodized oil intranodal lymphangiography with cyanoacrylate embolization for the treatment of lymphatic leak following robot-assisted pelvic resection.

## Materials and Methods

### Patient selection and inclusion and exclusion criteria

This study was conducted with Institutional Review Board approval and complied with the Health Insurance Portability and Accountability Act. Informed consent was not required. Patients referred to the interventional radiology service for diagnostic evaluation and therapeutic management of suspected lymphatic leak following pelvic resection were identified (*n* = 10). Patients in whom intranodal ethiodized oil lymphangiography with cyanoacrylate embolization was undertaken were selected (*n* = 4).

### Variables and outcome metrics

The electronic health record (EHR) was retrospectively reviewed for patient demographics, urologic procedural details, physical manifestations of postoperative lymphatic leak, conservative management methods (Table 1), details of the lymphangiography and embolization procedures, evidence of complications, and follow-up. Time of postoperative symptomatic presentation was recorded as the date of the first EHR report detailing physical evidence of lymphatic fluid accumulation. The quantity of cyanoacrylate injected was calculated as the total volume of embolic agent mixture multiplied by cyanoacrylate percent composition.

**Table 1.** Patient Characteristics of Urologic Surgery Patients with Postoperative Lymphatic Leak

*Age (years)/sex*	*Urologic surgery*	*Surgical indication*	*Physical signs of lymphatic leak*	*Fluid collection volume/size*	*Treatment before INL*	*Time from surgery to presentation (days)*	*Time from presentation to INL (days)*
75/M	Cystoprostatectomy with ileal conduit, b/l extended PLND	Urothelial carcinoma	Lymphatic ascites	3200 mL	Four paracenteses	27	21
84/M	Cystoprostatectomy with ileal conduit, b/l extended PLND	Urothelial carcinoma	Lymphatic ascites	2600 mL	Paracentesis	24	14
50/M	Prostatectomy, b/l standard PLND	Prostatic carcinoma	Left-sided pelvic lymphocele	3 cm	Lymphocele aspiration, sclerotherapy, and drain placement	54	77
65/M	Prostatectomy, b/l standard PLND	Prostatic carcinoma	b/l pelvic lymphoceles	20 mL	Lymphocele drain placement	3	117

INL = intranodal lymphangiography; M = male; b/l = bilateral; PLND = pelvic lymph node dissection.

Reported outcome metrics included the (1) technical success of ethiodized oil intranodal lymphangiography, (2) technical success of intranodal cyanoacrylate embolization, and (3) procedural clinical success (Table 2). Technical success of lymphangiography and embolization, respectively, was defined as uneventful cannulation of the inguinal lymph nodes with injected agent opacification of the lymphatics to the level of the retroperitoneal lymphatics extending beyond the site of identified extravasation. The procedure was considered clinically effective if there was sustained reduction in the volume of lymph accumulation. Follow-up time was calculated as the number of days from last lymphatic embolization to most recent medical evaluation by a healthcare provider, during which a comprehensive history and physical examination was performed.

**Table 2.** Procedural Success and Outcomes of Intranodal Lymphangiography and Direct Cyanoacrylate (*n*-BCA) Embolization

	*Initial lymphangiography*					*Repeat lymphangiography*				
*Age (years)/sex*	*Technical success*	*Intervention*	*Clinical success*	*Clinical picture*	*Time to repeat intervention (days)*	*Technical success*	*Intervention*	*Clinical success*	*Complications*	*Clinical picture*
75/M	Yes	INE with *n*-BCA	No	Recurrent ascites requiring four paracenteses	26	Yes	INE with *n*-BCA	Yes	None	Four paracenteses of diminishing volume until resolution
84/M	Yes	INE with *n*-BCA	Yes	Ascites resolution					None	
50/M	Yes	INE with *n*-BCA and lymphocele sclerotherapy	No	Persistent high-output lymphocele fluid production	9	Yes	INE with *n*-BCA	Yes	None	Lymphocele resolution
65/M	Yes	INE with *n*-BCA and lymphocele sclerotherapy	Yes	Lymphocele resolution					None	

*n*-BCA = *n*-butyl cyanoacrylate; INE = intranodal embolization; M = male.

### Patient demographics

Four adult males were identified as having undergone ethiodized oil intranodal lymphangiography with cyanoacrylate embolization for a lymphatic leak following urologic surgery. Patients underwent robot-assisted laparoscopic urologic surgery for resection of genitourinary malignancies at a mean age of 68.7 ± 14.3 years (Table 1). Urologic procedures were either cystoprostatectomy with ileal conduit and bilateral extended pelvic lymph node dissection for muscle-invasive urothelial carcinoma (*n* = 2) or prostatectomy with bilateral standard pelvic lymph node dissection for prostate carcinoma (*n* = 2).

Patients presented with physical signs suspicious for lymphatic leak at an average of 27.0 ± 20.9 days postoperatively. Signs included increasing abdominal girth (*n* = 2), persistent, high-volume drain output (*n* = 1), or infectious symptomatology (*n* = 1). Diagnostic evaluation with cross-sectional imaging and aspirated fluid evaluation identified lymphatic ascites (fluid triglycerides >131 mg/dL on all paracenteses performed, *n* = 2) and pelvic lymphoceles (*n* = 2) as outlined in Table 1. Patients with ascites required up to four therapeutic paracenteses before intranodal intervention.

### Intranodal lymphangiography and embolization techniques

**FIG. 1.**
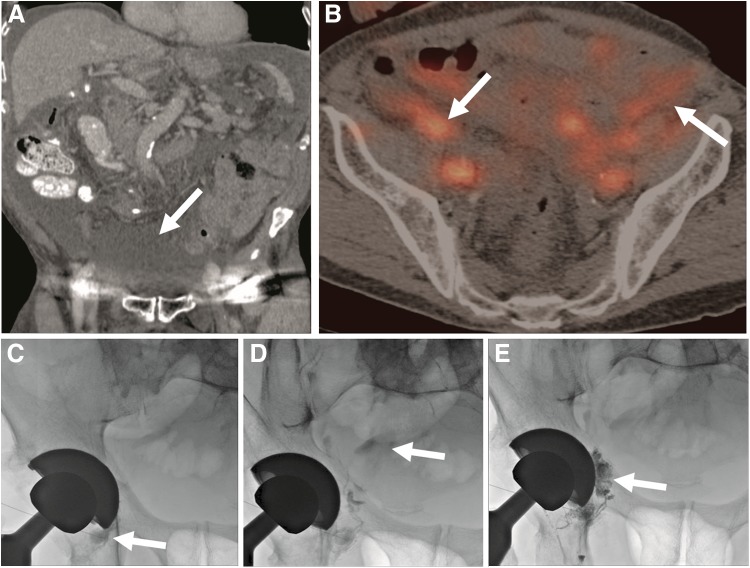
Eighty-four-year-old male with a history of muscle-invasive bladder cancer status post-cystoprostatectomy with ileal conduit. The patient presented with recurrent abdominal distension and lymphatic ascites. **(A)** Coronal computed tomography image of the abdomen demonstrated large volume ascites (*arrow*). **(B)** Nuclear medicine SPECT/CT Tc99m sulfur colloid lymphoscintigraphy demonstrated progressive pooling of radiotracer within the pelvis (*arrows*), compatible with a lymphatic leak. **(C)** A 25-gauge spinal needle was inserted into a right inguinal lymph node (*arrow*) and pelvic lymphangiography was performed. **(D)** With continued injection of contrast, extravasation was seen emanating from a right pelvic lymphatic vessel with pooling in the right hemipelvis (*arrow*). **(E)** The needle was subsequently primed with 5% dextrose. *n*-BCA glue was mixed with ethiodized oil (1 mL cyanoacrylate: 4 mL ethiodized oil) and was injected, thereby embolizing the leak (*arrow*). The patient's ascites subsequently resolved. CT, computed tomography; SPECT, single-photon emission computed tomography. *n*-BCA, *n*-butyl cyanoacrylate.

**FIG. 2.**
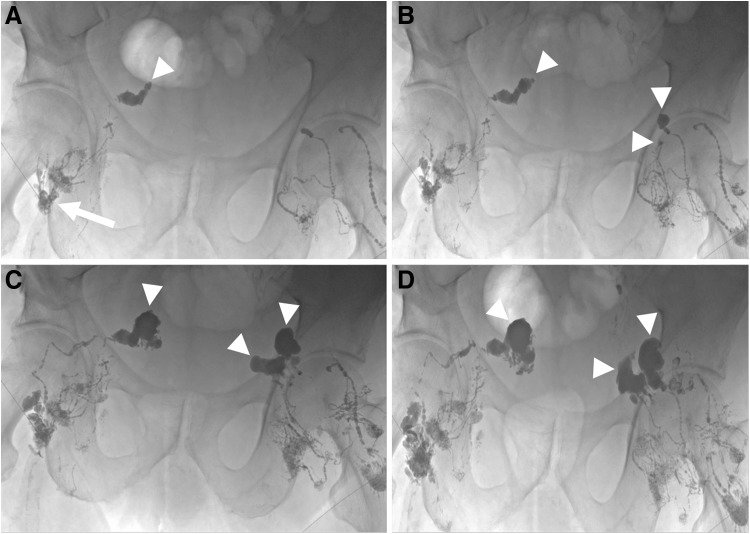
Seventy-five-year-old male with muscle-invasive bladder cancer status post-cystoprostatectomy with ileal conduit. The patient presented with large volume ascites. **(A)** A 25-gauge spinal needle was inserted into a right inguinal lymph node (*arrow*) and lymphangiography was performed using ethiodized oil. There was extravasation secondary to disrupted lymphatics in the right hemipelvis (*arrowhead*). **(B)** Bilateral intranodal pelvic lymphangiography demonstrated multifocal bilateral lymphatic disruptions (*arrowheads*). **(C)** Both needles were subsequently primed with 5% dextrose and cyanoacrylate glue embolization was performed (*arrowheads*) using *n*-BCA mixed with ethiodized oil (2 mL cyanoacrylate: 5 mL ethiodized oil). **(D)** Progressively more dense embolic agent was seen extending into the areas of lymphatic disruption (*arrowheads*) bilaterally thereby embolizing the leaks. The patient's ascites subsequently resolved.

Intranodal ethiodized oil lymphangiography and cyanoacrylate embolization techniques have been previously described^5,7^ and are shown in Figures 1 and 2. All patients were evaluated by an attending interventional radiologist during inpatient consultation before the procedure. Procedures were performed under moderate sedation with intravenous midazolam and fentanyl or general anesthesia administered by a certified registered nurse anesthetist or attending anesthesiologist.

Sterile preparation and draping of the inguinal region and pelvis were performed. Using ultrasound guidance, bilateral inguinal lymph nodes were accessed using 25-gauge spinal needles (Becton Dickinson, Franklin Lakes, NJ). Ethiodized oil (Lipiodol Ultra-Fluide; Guerbet, Villepinte, France) was injected directly into the accessed node with intermittent fluoroscopic observation and filling of the retroperitoneal lymphatics was assessed.

After opacification of the retroperitoneal lymphatics and identification of leak, the needle was flushed with 5% dextrose in water to limit premature, intraluminal glue polymerization. *n*-Butyl cyanoacrylate (TRUFILL; Codman Neurovascular, Raynham, MA) was diluted in ethiodized oil and the embolic agent was injected directly into the accessed lymph node. All needles were subsequently removed.

### Statistical analysis

Summary statistics, including percentages, means, ranges, and standard deviations, were computed using STATA software (version 15; StataCorp, College Station, TX).

## Results

### Ethiodized oil lymphangiography and cyanoacrylate glue embolization

Patients underwent ethiodized oil intranodal lymphangiography at a mean of 84.3 ± 48.0 (range: 38–131) days after pelvic resection and a mean of 57.3 ± 48.8 (range: 14–117) days following initial medical documentation of physical signs consistent with a lymphatic leak.

### Ethiodized oil lymphangiography and cyanoacrylate glue embolization technical success

In four patients, a total of six ethiodized oil intranodal lymphangiograms were performed, two procedures representing repeat interventions. Inguinal lymph node catheterization and ethiodized oil lymphatic system opacification was technically effective in all procedures (*n* = 6). Specifically, the inguinal, pelvic, and retroperitoneal lymphatic vessels were opacified to the level of the cisterna chyli from ethiodized oil injection into an inguinal lymph node. A mean of 5.2 ± 2.0 mL of ethiodized oil was injected into any given node.

Via the same 25-gauge needle used for ethiodized oil intranodal lymphangiography, a total of six cyanoacrylate glue embolization procedures were performed, two being repeated interventions. Intranodal injection of cyanoacrylate was technically effective in all procedures (*n* = 6). Cyanoacrylate was diluted to 24.2% and 51.7% in ethiodized oil for initial and repeat interventions, respectively. The embolic agent solution was injected until the lymphatic system was opacified beyond the site of contrast extravasation as identified on diagnostic lymphangiography. This injection volume resulted in a mean total cyanoacrylate instillation of 0.44 mL for first time embolizations and 0.52 mL of cyanoacrylate for repeat procedures.

### Clinical success

Primary clinical success, defined as a sustained reduction in the volume of lymph accumulation was achieved in 50% (*n* = 2/4) of patients after first intervention and in all (*n* = 4/4; 100%) patients following repeat intervention (Table 2). Lymphatic fluid accumulation resolved over time and remained absent as of most recent follow-up at a mean of 134.7 ± 79.2 days (range: 59–248 days) following endolymphatic embolization.

### Complications

No minor or major complications, morbidity, or mortality were associated with procedures. Specifically, no tissue necrosis, embolism, hypersensitivity reactions, or other complications were reported.

## Discussion

Abdominal, pelvic, and retroperitoneal lymphatic vessels are prone to disruption during genitourinary surgery. Diagnostic evaluation of lymphatic system disruption with ethiodized oil intranodal lymphangiography delineates the location and extent of a leak and confirms that disrupted sites are fed by the accessed node. At this point, as described, cyanoacrylate glue may be injected through the same access needle. There was excellent technical success for intranodal lymphangiography with cyanoacrylate embolization, with the potential to detect lymphatic injuries to the level of the cisterna chyli. Primary clinical success was seen in half of the patient sample (*n* = 2/4). Repeat embolization was required in two patients which was ultimately clinically effective. Overall, the clinical success with repeat embolization was 100% (*n* = 4/4).

The direct intranodal technique described here is more minimally invasive than previously reported techniques.^4–6^ Ethiodized oil intranodal lymphangiography plays an important role in the anatomical visualization of the site and extent of lymphatic leakage and, with the use of ethiodized oil as contrast, has some therapeutic potential for occluding lymphatic leaks. While clinical success rates for ethiodized oil instillation alone have been reportedly as high as 71.4% for the occlusion of low output leaks,^2^ therapeutic efficacy in cases with lymphatic drainage rates greater than 500 mL/day decreases to 35%.^3^ Additional percutaneous embolization approaches, including “closest upstream lymph node” and “direct upstream lymphatic vessel” embolization, have been used with good clinical efficacy.^4–6^ These techniques require gaining additional access more proximal to the site of demonstrated vessel disruption that may not always be feasible given the small size and fragility of lymphatic vessels. Access and embolization of multiple lymph nodes with cyanoacrylate glue may be necessary depending on the extent of lymphatic disruption.

There are several technical considerations that should be taken into account when implementing this embolization approach. First, if there is a concern for premature polymerization of the cyanoacrylate glue in the proximal lymphatic system before reaching the point of disruption, care should be taken to further appropriately dilute the injected cyanoacrylate glue to extend polymerization time. Second, the accessed node closest to the site of disruption is most likely to effectively occlude the site of leakage. Third, as mentioned in previous reports,^4^ local ethiodized oil pooling at the site of failed access may make subsequent access to lymph nodes in the local vicinity challenging. If the efferent lymphatic system is not immediately opacified with ethiodized oil, injection should be stopped.

There are several avenues for future investigation with this approach. Prospective assessment of the therapeutic efficacy of intranodal lymphangiography and embolization may identify characteristics associated with first intervention clinical success. Relevant, potentially predictive variables include time from urologic surgery to intranodal lymphangiography, time from presentation with physical evidence of lymphatic leak to intranodal lymphangiography, anatomic location and extent of lymphadenectomy,^8^ patient age and body mass index,^8^ and fluid collection size. Identification of specific characteristics associated with first intervention clinical success using this direct intranodal technique may obviate the use of unnecessary, more invasive treatment options.

This report has several limitations. It is retrospective and describes a small case series collected from a single institution. Choice of interventions was selected at provider discretion, thus introducing ascertainment bias. This study was underpowered to identify anatomic characteristics associated with, and potentially predictive of, technical and therapeutic success using this technique.

## Conclusion

Intranodal ethiodized oil lymphangiography with direct cyanoacrylate embolization is an effective technique for the management of postoperative abdominal and pelvic lymphatic injuries in urologic surgery patients.

## Abbreviations Used

EHR: electronic health record
*n*-BCA: *n*-butyl cyanoacrylate
